# Convulsant bicuculline modifies CNS muscarinic receptor affinity

**DOI:** 10.1186/1471-2202-7-32

**Published:** 2006-04-17

**Authors:** Patricia G Schneider, Georgina Rodríguez de Lores Arnaiz

**Affiliations:** 1 Instituto de Biología Celular y Neurociencias "Prof. E. De Robertis", Facultad de Medicina, Universidad de Buenos Aires, Paraguay 2155, 1121-Buenos Aires, Argentina; 2 Cátedra de Farmacología, Facultad de Farmacia y Bioquímica, Universidad de Buenos Aires, Junin 956, 1121-Buenos Aires, Argentina

## Abstract

**Background:**

Previous work from this laboratory has shown that the administration of the convulsant drug 3-mercaptopropionic acid (MP), a GAD inhibitor, modifies not only GABA synthesis but also binding of the antagonist [^3^H]-quinuclidinyl benzilate ([^3^H]-QNB) to central muscarinic receptors, an effect due to an increase in affinity without modifications in binding site number. The cholinergic system has been implicated in several experimental epilepsy models and the ability of acetylcholine to regulate neuronal excitability in the neocortex is well known. To study the potential relationship between GABAergic and cholinergic systems with seizure activity, we analyzed the muscarinic receptor after inducing seizure by bicuculline (BIC), known to antagonize the GABA-A postsynaptic receptor subtype.

**Results:**

We analyzed binding of muscarinic antagonist [^3^H]-QNB to rat CNS membranes after i.p. administration of BIC at subconvulsant (1.0 mg/kg) and convulsant (7.5 mg/kg) doses. Subconvulsant BIC dose failed to develop seizures but produced binding alteration in the cerebellum and hippocampus with roughly 40% increase and 10% decrease, respectively. After convulsant BIC dose, which invariably led to generalized tonic-clonic seizures, binding increased 36% and 15% to cerebellar and striatal membranes respectively, but decreased 12% to hippocampal membranes. Kd value was accordingly modified: with the subconvulsant dose it decreased 27% in cerebellum whereas it increased 61% in hippocampus; with the convulsant dose, Kd value decreased 33% in cerebellum but increased 85% in hippocampus. No change in receptor number site was found, and Hill number was invariably close to unity.

**Conclusion:**

Results indicate dissimilar central nervous system area susceptibility of muscarinic receptor to BIC. Ligand binding was modified not only by a convulsant BIC dose but also by a subconvulsant dose, indicating that changes are not attributable to the seizure process itself. Findings support the notion that the muscarinic receptors play a major role in experimental epilepsy and provide a new example of differential neuronal plasticity.

## Background

An imbalance between inhibition and excitation in the CNS may lead to seizures that involve the synchronous and repetitive discharges of a large group of neurons.

Convulsant drug administration has proven to be effective and therefore a useful tool to study experimental epilepsy, since it produces behavioral modifications concomitant with marked neurochemical changes. Acetylcholine is an essential neurotransmitter/neuromodulator in several experimental epilepsy disorders [1], and its ability to regulate neuronal excitability in neocortex is well known [2]. Interestingly, the anticonvulsant effect mediated by carbachol microinjection in the nucleus reticularis pontis oralis appears to be mediated by muscarinic receptors [3].

GABA is the major central inhibitory neurotransmitter, and it is not surprising, therefore, that it has been involved in epileptic activity genesis [4-6]. The biosynthesis of GABA requires the activity of glutamic acid decarboxylase (GAD), a cytosolic enzyme that is found in neurons where GABA is a neurotransmitter.

Previous work from this laboratory has shown that the administration of the convulsant drug 3-mercaptopropionic acid (MP), a GAD inhibitor [7], modifies not only GABA synthesis [8,9] but also binding of antagonist [^3^H]-quinuclidinyl benzilate ([^3^H]-QNB) to central muscarinic receptors, an effect due to an increase in affinity without modifications in binding site number [10-12].

To study potential relationship between GABAergic and cholinergic systems with seizure activity, we analyzed the muscarinic receptor after inducing seizure by bicuculline (BIC), known to antagonize GABA-A postsynaptic receptor subtype [13]. Subconvulsant and convulsant BIC doses were administered to rats and [^3^H]-QNB binding to cortical, striatal, cerebellar and hippocampal membranes assayed. As significant changes were found in cerebellum and hippocampus (increase and decrease, respectively), such membranes were chosen to perform saturation studies and Scatchard analysis, to observe significant alteration in binding affinitty.

## Results

**Figure 1:**
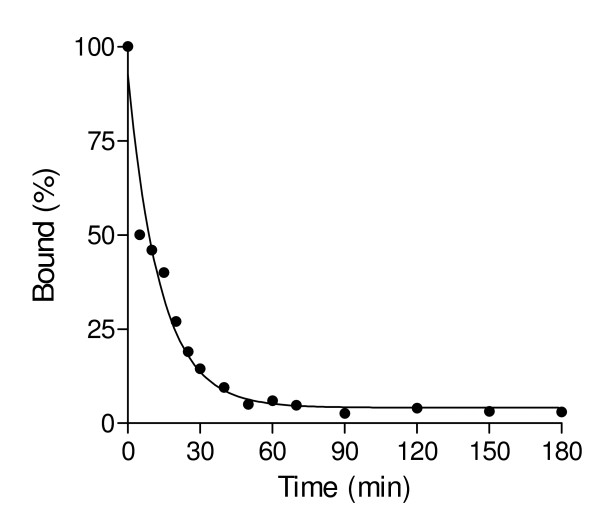
Dissociation kinetic for [^3^H]-QNB binding to rat control cerebral cortex membranes. Assay was performed at 30°C with 0.5 nM [^3^H]-QNB. At different time intervals (1 to 180 min), 3 ml samples were filtered and washed. Values represent a single experiment performed in duplicate.

Kinetic studies in experiments of association-dissociation binding for [^3^H]-QNB were performed in control rat cerebral cortex membranes. The time course was analyzed between 1 and 180 min at 30°C (Fig. 1). Binding parameters obtained (mean values ± S. D., n = 3) were: k_obs _(min^-1^) = 0.08 ± 0.02; k_on _(min^-1 ^.nM^-1^) = 0.8 ± 0.2; k_off _= 0.03 ± 0.004; K_d _= k_off/_k_on _(nM) = 0.04 and K_d_' (nM) = 0.136 ± 0.050. The discrepancy in the values of the K_d_' and the K_d _obtained in the equilibrium and kinetic experiments, respectively, is probably a consequence of the high receptor concentration used in the binding assays [14]. Since binding equilibrium was attained at 40 – 50 min, further experiments were carried out at 60 min incubation.

To examine potential changes in muscarinic receptors, [^3^H]-QNB binding to rat CNS membranes isolated from control and BIC treated animals was assayed.

### Effect of a subconvulsant BIC dose

Intraperitoneal rat administration of 1.0 mg/kg BIC failed to develop seizures but slight abnormal behaviour changes as sniffing and intensive washing movements were observed during 5–10 minutes, when animals recovered normal behavior.

Significant changes in [^3^H]-QNB binding in cerebellum and hippocampus were found. In cerebellum, an increase of roughly 40% was recorded, since ligand binding in pmol per mg protein was 0.50 in BIC treated *versus *0.35 in control group. In hippocampus, 10% decrease was obtained, with values of 1.35 in BIC treated *versus *1.50 in control group. No significant changes were found in cortical or striatal membranes (Table 1).

### Effect of a convulsant BIC dose

Intraperitoneal rat injection of 7.5 mg/kg BIC resulted in the development of generalized tonic-clonic seizures. As a rule, after 90–120 sec latency, rats suddenly ran amok (during 10–30 sec), then became motionless, regained tonus (30–45 sec), and ended up with a four-limb clonic phase (90–150 sec). Present experiments were performed with animals decapitated at the onset of seizure, during the running stage.

At seizure stage, [^3^H]-QNB binding to cerebellar membranes exhibited 36% increase, since ligand binding in pmol per mg protein was 0.46 in BIC treated *versus *0.34 in control group. Again, a 12% decrease was observed in hippocampus, with data, in pmol per mg protein, of 1.36 in BIC *versus *1.50 in control group. [^3^H]-QNB binding to striatal membranes increased 15% with values of 2.27 in BIC treated *versus *1.98 in control group; no significant changes were found in cortical membranes (Table 2).

### Saturation studies

**Figure 2:**
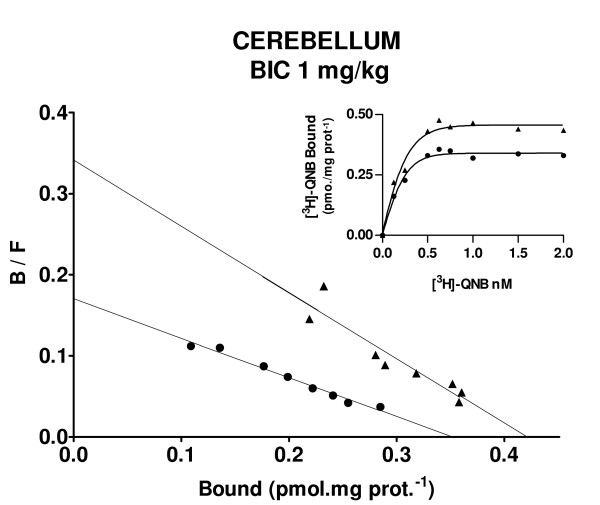
Scatchard plots for [^3^H]-QNB binding to rat cerebellar membranes at equilibrium after administration of a subconvulsant BIC dose (1.0 mg/kg). Results are from a single experiment representative of a set of three, each performed in duplicate. Inset, saturation curves. (●) Control; (▲) BIC subconvulsive.

**Figure 3:**
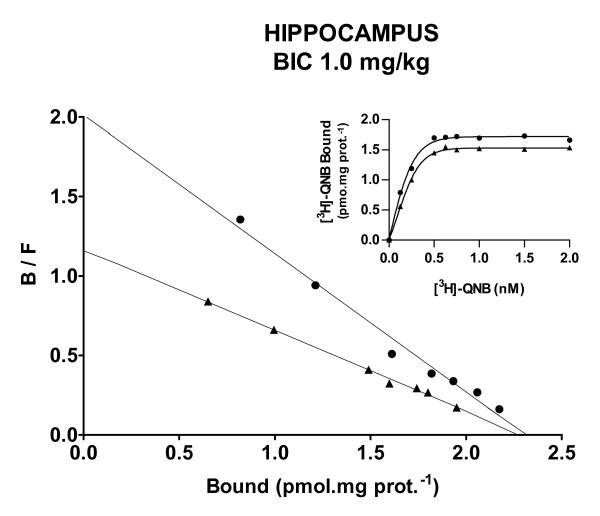
Scatchard plots for [^3^H]-QNB binding to rat hippocampal membranes at equilibrium after administration of a subconvulsant BIC dose (1.0 mg/kg). Results are from a single experiment representative of a set of three, each performed in duplicate. Inset, saturation curves. (●) Control; (▲) BIC subconvulsive.

**Figure 4:**
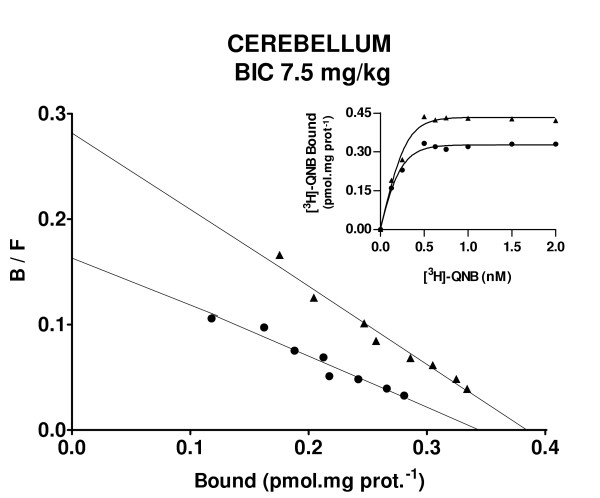
Scatchard plots for [^3^H]-QNB binding to rat cerebellar membranes at equilibrium after administration of a convulsant BIC dose (7.5 mg/kg). Results are from a single experiment representative of a set of three, each performed in duplicate. Inset, saturation curves. (●) Control; (▲) BIC seizure.

**Figure 5:**
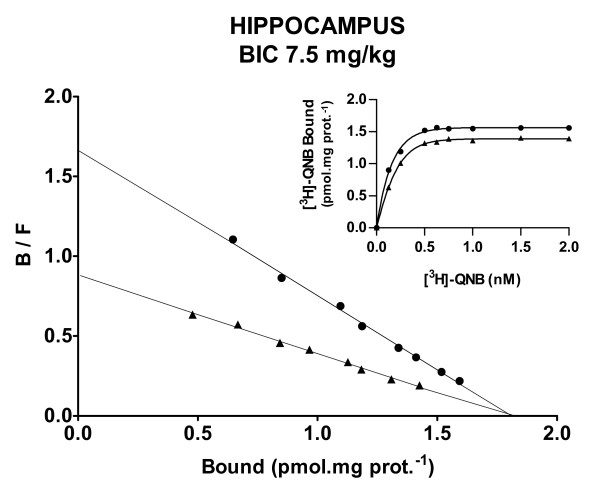
Scatchard plots for [^3^H]-QNB binding to rat hippocampal membranes at equilibrium after administration of a convulsant BIC dose (7.5 mg/kg). Results are from a single experiment representative of a set of three, each performed in duplicate. Inset, saturation curves. (●) Control; (▲) BIC seizure.

In order to determine whether binding changes observed in cerebellum and hippocampus were due to modifications in affinity and/or site number, [^3^H]-QNB binding was studied at variable ligand concentrations. Saturation values were attained with 0.50–1.00 nM ligand in membranes after subconvulsant and convulsant BIC doses as well as in control membranes (Figs. 2, 3, 4, 5).

Scatchard analysis of [^3^H]-QNB binding data recorded at equilibrium disclosed a significant decrease (27%) in cerebellum K_d _value in membranes obtained from rats treated with 1.0 mg/kg BIC dose; on the contrary, hippocampus K_d _value increased 61%. No change in receptor site number was recorded in either area. Hill number was close to unity and remained unaltered in both cerebellar and hippocampal membranes (Table 3).

At seizure stage, K_d _value decreased 33% in cerebellum, but increased 85% in hippocampus. No significant change in B_max _was found either in cerebellar or hippocampal membranes. Here again, Hill number was close to unity and remained unaltered in either case (Table 4).

Equilibrium binding data for [^3^H]-QNB recorded at several ligand concentrations were analyzed by a computer to disclose whether a single or two population sites were operative; it was observed that the best fit indicated a single population site.

## Discussion

To analyze muscarinic receptor under GABA-A receptor blockade, [^3^H]-QNB binding to rat CNS membranes after the administration of subconvulsant and convulsant BIC doses was studied. The subconvulsant BIC dose produced significant changes in [^3^H]-QNB binding, with an increase in cerebellar but a decrease in hippocampal membranes, without alterations in either striatal or cortical membranes. With the convulsant BIC dose, binding increased to cerebellar and striatal membranes but decreased to hippocampal membranes whereas it remained unaltered to cortical membranes. Whenever alterations in binding were recorded, they were invariably due to affinity changes alone, since site number remained constant. Scatchard plots obtained at equilibrium rendered linear profiles and Hill number was close to unity, suggesting that [^3^H]-QNB binds to a homogeneous site population in all cases.

On the basis of studies performed in diverse animal models, the concept that epileptic episodes may be caused by imbalance between inhibition and excitation inputs has been advanced [1,15-17]. Synaptic inhibition is a regulatory and crucial mechanism which limits the generation of action potential in neurons.

Glutamatergic NMDA receptors are known to exert a role in seizure [18,19]; however, the possible involvement of other receptor types, *i. e*., muscarinic receptors, has been advanced [4,20-22], among which M_1 _is the only subtype mediating pilocarpine-induced seizure [23,24].

Several lines of evidence point to a relationship between cholinergic muscarinic activation, GABA system and seizure activity. Previous work from this laboratory has shown that convulsant MP modifies GABA system by decreasing GAD activity and GABA levels as well as alterating of cerebellar Purkinje cell morphology [8,9]. With a convulsant MP dose, [^3^H]-QNB binding to muscarinic receptors is enhanced in CNS, an effect due to an increase in affinity without changes in binding site number [10,11]. However, a subconvulsant MP dose fails to induce any change [10-12]. Herein it is shown that BIC administration either at subconvulsant or convulsant dose, is able to modify [^3^H]-QNB binding to muscarinic receptor. Therefore, the explanation that alteration of muscarinic receptor is a consequence of seizure process seems untenable.

The increase in ligand binding to striatal and cerebellar membranes with convulsant BIC dose may well indicate the activation of cholinergic excitatory circuits. These findings are consistent with the observation that muscarinic receptor activation during BIC blockade of GABA-A-mediated potentials induces synchronous epileptiform activity in immature rat CNS [25,26].

Herein we recorded a small but significant decrease in binding in hippocampal membranes obtained with convulsant and subconvulsant BIC doses, an expected result since after short seizure hippocampus undergoes cellular, functional and structural alterations following the administration of convulsant drugs [27-29]. Interestingly, in this epileptogenic area, interactions between cholinergic and GABAergic systems have been doccumented [16,30-32]. Present results in hippocampus differ from those recorded with convulsant MP [10-12], a finding which may receive an explanation in the different mechanism of action of BIC *versus *MP.

As regards results recorded in striatum, where binding changes were observed only after BIC convulsant dose, it should be recalled that this area participates in motor activities. In this connection, muscarinic receptor alteration in striatum is observed after high doses of muscarinic agonist pilocarpine [33].

Among CNS studied areas, cerebellum presents the lowest density of muscarinic receptor sites; however, the greatest changes in [^3^H]-QNB binding after BIC administration were recorded in this area, attributable to cerebellum participation in motor activity, markedly altered during convulsive activity.

Normal neurophysiological functions as adaptation, inhibition and facilitation, among others, are associated with plasticity, participating in compensatory processes by which the CNS adapts to pathological conditions, exposure to drugs and neuronal damage and loss [34]. Neuronal plasticity evidences the response of a circuit, a neurotransmitter or a receptor that is modified as a result of different factors or altered processes. Herein it is shown that muscarinic receptor changed promptly after stimulus (BIC administration), a finding in line with the observation that this receptor is enhanced in the human epileptic focus at early times following seizures through activity-dependent mechanisms [35].

On comparing previous results obtained with MP *versus *present findings with BIC, a differential response of muscarinic receptor is evident according to GABA system site alteration, that is, when GAD activity is inhibited or GABA-A receptor antagonized.

To sum up, results indicate that: 1) seizure activity itself is not the sole mechanism liable to produce a change in muscarinic receptors, since a subconvulsant BIC dose also induces modifications; 2) there is dissimilar area susceptibility for muscarinic receptor to BIC; and 3) muscarinic receptor response to convulsant drugs (MP or BIC) support differential neuronal plasticity. Findings support the notion that muscarinic receptors play a major role in experimental epilepsy,

## Conclusion

Results indicate dissimilar central nervous system area susceptibility of muscarinic receptor to BIC. Ligand binding was modified not only by a convulsant BIC dose but also by a subconvulsant dose, indicating that changes are not attributable to seizure process itself. Findings support the notion that the muscarinic receptors play a major role in experimental epilepsy and provide a new example of differential neuronal plasticity.

## Methods

### Animals and drug treatment

Young adult male Wistar rats (25 days old) weighing 100–150 g were used. Animals caged in groups of five were housed at constant temperature (20–23°C) and maintained at least one week in a 12 h light-dark cycle (from 9.00 a.m. to 9.00 p.m.) with free access to food and water. Rats were injected i.p. between 9.30 and 11.30 a.m. with fresh BIC solutions to reach 1.0 and 7.5 mg/kg doses. BIC (Sigma, St. Louis, MO) was dissolved in 0.1 N HCl, brought to pH 5 with 0.1 N NaOH and immediately injected. All studies described were conducted in accordance with the Guide for Care and Use of Laboratory Animals provided by the National Institutes of Health, USA.

### Experimental groups

Three experimental groups were used: control, subconvulsant, and convulsant. Animals in the control group received no treatment. In the subconvulsant group, rats were injected with 1 mg/kg BIC dose, a dose which failed to induce seizure or behavioral modifications. In the convulsant group, animals were injected with 7.5 mg/kg BIC, a dose that produced generalized tonic-clonic seizures. For each BIC dose, lots of 5 rats each time with their corresponding controls were used.

Rats were decapitated at 30 min after injection (subconvulsant stage or condition) or at the onset of seizure (convulsant stage or condition), respectively. Each condition was repeated 3–6 times.

Since saline administration produces no change in any of the areas studied [11], uninjected rats were used as controls.

### Membrane preparations

For each experimental condition cerebellum, hippocampus, cerebral cortex and striatum from five animals were harvested and separately pooled. Tissues were rapidly homogenized at 10% w/v, except for cerebral cortex at 4% w/v, in 0.32 M sucrose neutralized with Tris base solution (0.4 mM Tris final concentration) in a Teflon glass Potter-Elvehjem homogenizer.

Homogenates were centrifuged at 900 *g *for 10 min and pellets discarded; resulting supernatants were diluted with 0.16 M sucrose to achieve a final concentration of 0.25 M sucrose, centrifuged at 100,000 *g *for 30 min and membrane pellets stored at -70°C until use.

### [^3^H]-QNB binding assay

[^3^H]-QNB binding was determined according to the method described by Yamamura and Snyder [36] with slight modifications. Membrane pellets were resuspended and later diluted in 50 mM sodium, potassium phosphate buffer (pH 7.4) to reach 0.1 mg protein/ml concentration. Triplicate membrane aliquots were incubated (2 ml final volume) at 30°C in the presence of 0.5 nM L- [^3^H]-QNB (S- [^3^H]-QNB enantiomer), Du Pont Corp. New England Nuclear, Boston, MA, USA, specific activity 14,443 GBq/mmol), with or without 5 μM atropine sulfate. Incubation proceeded for 60 min, because [^3^H]-QNB binding reached equilibrium after 45 min incubation, in agreement with data from the literature [37-39].

After incubation, 3 ml of ice-cold sodium, potassium phosphate buffer were added and samples vacuum-filtered through Whatman GF/B glass disks. Filters were washed twice with 3 ml of ice-cold buffer, placed in plastic vials and dried overnight at 70°C. To each vial, 3 ml of 0.4% 2,5-diphenyl-oxazole in toluene were added and radioactivity quantified in a liquid scintillation counter.

Specific binding was calculated as the difference between the binding in the absence and presence of 5 μM atropine sulfate, and represented *ca *90% of total binding.

Results averaged were obtained with different membranes isolated from tissue pooled (from 5 rats each group). The whole experiment (BIC administration, tissue harvesting, membrane preparation and binding assay in triplicate) was carried out in 3 or 6 different occasions.

For saturation studies, duplicate membrane samples were incubated in the presence of [^3^H]-QNB ranging from 0.125 to 2.00 nM concentration and processed as described above. The same basic filtration assay was used in experiments in which the kinetics of binding were investigated. For measurement of association rates, cerebral cortex membranes were incubated at 0.1 mg/ml protein concentration, in the presence of 0.5 nM L- [^3^H]-QNB. At different time intervals extending over 180 min, 3 ml samples were removed and filtered as indicated above. Additional samples were incubated in the presence of 5 μM atropine sulfate to assay nonspecific binding. The measurement of dissociation rates was performed in a similar way on membranes that had reached equilibrium binding after incubation for 60 min in the presence of 0.5 nM L- [^3^H]-QNB.

Protein was assayed according to Lowry et al. [40] using bovine serum albumin as standard.

### Data analysis

Differences in mean values between groups were evaluated by Student's-*t *test. Significance levels were set at *P<*0.05.

For saturation assays, non-linear regression of the data were processed using EBDA program (G. A. Mc Pherson 1983 V 2.0). Scatchard transformation of the data obtained at equilibrium was employed to show whether more than one receptor population was operative.

## Authors' contributions

PGS and GRLA participated conjointly in the design, performing assays and writting the manuscript. The authors read and approved the final manuscript.

## Figures and Tables

**Table 1:** Effect of a subconvulsant BIC dose (1.0 mg/kg, i.p.) on [^3^H]-QNB binding to membranes isolated from four brain areas.

Area	[^3^H]-QNB binding		
	
	Condition		
	Control pmol.mg protein^-1^	Subconvulsive pmol.mg protein^-1^	Δ (%)
Cerebellum	0.35 ± 0.04 (3)	0.50 ± 0.04** (3)	+ 43
Hippocampus	1.50 ± 0.03 (3)	1.35 ± 0.03* (3)	- 10
Striatum	1.98 ± 0.11 (3)	2.09 ± 0.05 (3)	+ 6
Cerebral cortex	1.35 ± 0.04 (3)	1.44 ± 0.14 (3)	+ 7

Values are means ± S.D. Figures in parentheses denote number of separate experiments performed in triplicate.

* *P *< 0.05, ** *P *< 0.01 (Student's-*t *test).

**Table 2:** Effect of a convulsant BIC dose (7.5 mg/kg, i.p.) on [^3^H]-QNB binding to membranes isolated from brain areas.

Area	[^3^H]-QNB binding		
	
	Condition		
	Control pmol.mg protein^-1^	Convulsive pmol.mg protein^-1^	Δ (%)
Cerebellum	0.34 ± 0.03 (6)	0.46 ± 0.03*** (6)	+ 36
Hippocampus	1.55 ± 0.07 (6)	1.36 ± 0.08** (6)	- 12
Striatum	1.98 ± 0.11 (3)	2.27 ± 0.14* (3)	+ 15
Cerebral cortex	1.35 ± 0.04 (3)	1.42 ± 0.04 (3)	+ 5

Values are means ± S.D. Figures in parentheses denote number of separate experiments performed in triplicate.

* *P *< 0.05, ** *P *< 0.01, *** *P *< 0.001 (Student's-*t *test).

**Table 3:** [^3^H]-QNB binding constants in cerebellar and hippocampal membranes after administration of a subconvulsant BIC dose (1.0 mg/kg).

Area	Condition	K_d _(pM)	Δ (%)	B_max _(pmol.mg prot^-1^)	N_H_
Cerebellum	Control	197.5 ± 6.0		0.36 ± 0.08	1.01 ± 0.02
	Subconvulsive	144.9 ± 5.5***	- 27	0.42 ± 0.07	0.98 ± 0.03
					
Hippocampus	Control	115.4 ± 5.8		2.31 ± 0.09	0.97 ± 0.04
	Subconvulsive	185.6 ± 3.9***	+ 61	2.38 ± 0.10	0.96 ± 0.02

For each experiment, cerebellum and hippocampus from five rats were pooled, membranes separated and [^3^H]-QNB binding assayed. Data from three experiments were processed to calculate constants. Results presented are mean values ± S.D.

*** *P *< 0.001 (Student's-*t *test).

**Table 4:** [^3^H]-QNB binding constants in cerebellar and hippocampal membranes after administration of a convulsant (7.5 mg/kg) BIC dose.

Area	Condition	K_d _(pM)	Δ (%)	B_max _(pmol.mg prot^-1^)	N_H_
Cerebellum	Control	200.1 ± 7.1		0.35 ± 0.01	1.00 ± 0.01
	Convulsive	133.3 ± 7.5***	- 33	0.42 ± 0.07	1.05 ± 0.02
					
Hippocampus	Control	108.8 ± 2.7		1.75 ± 0.08	1.01 ± 0.06
	Convulsive	185.6 ± 3.9***	+ 85	1.72 ± 0.11	0.98 ± 0.02

For each experiment, cerebellum and hippocampus from five rats were pooled, membranes separated and [^3^H]-QNB binding assayed. Data from three experiments were processed to calculate constants. Results presented are mean values ± S.D.

*** *P *< 0.001 (Student-*t *test).
